# 7. Clinical outcomes and epidemiological characteristics of bacteremia in the older population of Japan

**DOI:** 10.1093/ofid/ofab466.007

**Published:** 2021-12-04

**Authors:** Keiji Nakamura, Kayoko Hayakawa, Shinya Tsuzuki, Satoshi Ide, Hidetoshi Nomoto, Takato Nakamoto, Gen Yamada, Kei Yamamoto, Norio Ohmagari

**Affiliations:** 1 Disease Control and Prevention Center, National Center for Global Health and Medicine, Tokyo, Japan, Fukuoka, Fukuoka, Japan; 2 National Center for Global Health and Medicine Hospital, Shinjuku, Tokyo, Japan; 3 National Center for Global Health and Medicine, Shinjuku-ku, Tokyo, Japan

## Abstract

**Background:**

Japan is one of the most aging societies worldwide. Because older people are highly susceptible to infectious diseases, the characteristics and clinical consequences of bacteremia in this population need clarification.

**Methods:**

Patients aged ≥ 65 years with positive blood cultures were included in this study conducted between April 1, 2015 and March 31, 2018, and divided into three groups: pre-old (65–74 years), old (75–89 years), and super-old (≥90 years) according to the criteria of the Japanese Society of Geriatrics. They were also classified based on medical exposure: community-acquired (CA), healthcare-associated (HCA), and hospital-onset (HO). Parameters retrieved from medical records were used to compare each group using the chi-square test or Fisher’s exact test; factors related to mortality were identified using multivariate logistic regression analysis after controlling for the confounding effect of baseline characteristics and underlying diseases. The Bonferroni corrected P < 0.05 was deemed to be statistically significant.

**Results:**

Overall, 1716 cases of bacteremia were identified in 1415 patients. Of these, 505 cases (29.4%) were found to be due to contamination. Of the 1211 cases without contamination, 397 (32.8%) included pre-old, 658 (54.3%) included old, and 156 (12.9%) included super-old patients. HCA bacteremia increased with age, while HO bacteremia was most common in pre-old patients. *Escherichia coli* bacteremia was most common in super-old patients. While a central line-associated bloodstream infection was more common in pre-old patients, a urinary tract infection was more common in old and super-old patients. The 7-day mortality was 7.4%, 5.8%, and 14.2% in the pre-old, old, and super-old groups, respectively (P = 0.002). The 7-day mortality for CA, HCA, and HO bacteremia was 5.4%, 6.6%, and 9.5%, respectively (P > 0.05). Multivariate logistic regression showed that HO bacteremia (aOR: 1.76 [1.05–2.94], P = 0.028) and increasing age (aOR: 1.03 [1–1.06], P=0.038) are independent risk factors for 7-day mortality.

Table Comparison of characteristics of bacteremia among the pre-old, old, super-old groups, n (%)

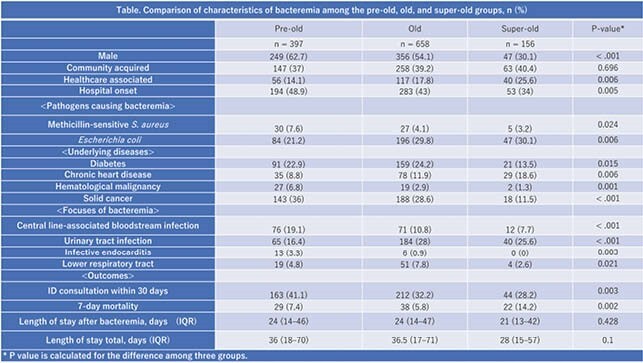

**Conclusion:**

The epidemiology of bacteremia differs among different older age groups; thus, these populations should not be treated as a single entity. A careful approach is needed for the optimal management of bacteremia in them.

**Disclosures:**

**All Authors**: No reported disclosures

